# Molecular characterization of mudskipper (*Boleophthalmus pectinirostris*) hypoxia-inducible factor-1α (HIF-1α) and analysis of its function in monocytes/macrophages

**DOI:** 10.1371/journal.pone.0177960

**Published:** 2017-05-24

**Authors:** Feng Guan, Xin-Jiang Lu, Chang-Hong Li, Jiong Chen

**Affiliations:** 1 Laboratory of Biochemistry and Molecular Biology, School of Marine Sciences, Ningbo University, Ningbo, China; 2 College of Biological and Environmental Sciences, Zhejiang Wanli University, Ningbo, China; 3 Collaborative Innovation Center for Zhejiang Marine High-efficiency and Healthy Aquaculture, Ningbo University, Ningbo, China; Kyung Hee University, REPUBLIC OF KOREA

## Abstract

Hypoxia-inducible factor-1α (HIF-1α) plays a critical role in immune and inflammatory responses and is important in controlling a variety of processes in monocytes and macrophages. However, very little information is available about the functions of HIF-1α in fish monocytes/macrophages (MO/MФ). In this study, the cDNA sequence of the mudskipper (*Boleophthalmus pectinirostris*) HIF-1α gene (BpHIF-1α) was determined. Sequence comparison and phylogenetic tree analysis showed that BpHIF-1α is clustered in the fish HIF-1α tree. Constitutive expression of BpHIF-1α mRNA was detected by real-time quantitative PCR in all tested tissues, and the expression was found to be dramatically increased in the skin, liver, spleen, and kidney after *Edwardsiella tarda* infection. In addition, hypoxia and infection induced the expression of the BpHIF-1α transcript and protein in MO/MФ, respectively. Hypoxia caused an increase in phagocytic and bactericidal capacity of mudskipper MO/MФ in a BpHIF-1α-dependent manner. BpHIF-1α induced an anti-inflammatory status in MO/MФ upon *E*. *tarda* infection and hypoxia. Therefore, BpHIF-1α may play a predominant role in the modulation of mudskipper MO/MФ function in the innate immune system.

## Introduction

Hypoxia is a common phenomenon in aquatic systems worldwide, and there is growing concern that hypoxia may affect aquatic animals [1]. Environmental hypoxia can modulate and compromise behavior and/or physiology, and manipulate the immune response in fish [2, 3]. Aquatic hypoxia induces changes in the fish immune system, involving cytokines, granulocytes, and macrophages [4–6]. Slight hypoxic stress can elevate non-specific immune responses in fish, but severe hypoxic challenge results in immunosuppression [7, 8]. Therefore, hypoxia is now considered as a potential regulator of immunity.

Hypoxia-inducible factor-1 (HIF-1) is a heterodimeric transcription factor, composed of two subunits (α and β), which regulate changes in gene expression when animals adapt to hypoxic conditions [9]. In majority of the cells, HIF-1 is primarily regulated through oxygen-dependent degradation of its HIF-1α-subunit and regulation of the transcriptional activity of the C-terminal transactivation domain [10–12]. HIF-1α is the master transcriptional regulator of cellular and developmental response to hypoxia) [13]. Non-hypoxic stimuli such as lipopolysaccharides (LPS) also up-regulate HIF-1α gene expression at both transcriptional and post-transcriptional levels in specific immune cells such as macrophages [14]. Exposure of sea bass to hypoxia results in a seven-fold increase of HIF-1α transcription factor in the liver [15]. Hypoxia elicits HIF-1α accumulation in teleost fish [16, 17]. In mice, hypoxia-induced HIF-1α is assumed to be involved in cytokine induction and causes an increase in phagocytosis and bactericidal activity in macrophages [18–21], which may be triggered by nuclear factor kappa B (NF-κB) and HIF signaling pathways [22, 23]. However, the effects of HIF-1α on the immune response of macrophages in fish are unknown.

Differences between the more terrestrial (i.e. exposed to desiccation and hypoxia) and the more aquatic mudskippers (*Boleophthalmus pectinirostris*) in HIF-1α regulation could be an interesting topic for future research [24]. *B*. *pectinirostris* is usually found in temperate-climate intertidal mudflats around the coast of Japan, Taiwan, and Southern China, where it is consumed as a culinary delicacy having high nutritional value. In this study, we identified the *B*. *pectinirostris* HIF-1α gene (BpHIF-1α). In addition, we measured the mRNA expression of BpHIF-1α in different tissues following bacterial infection and analyzed the effect of BpHIF-1α on monocytes/macrophages (MO/MФ).

## Materials and methods

### Fish rearing

Healthy mudskippers, weighing 30–40 g each, were purchased from a commercial Lulin market (N29°54′, E121°36′) in Ningbo city, China. Fish were transferred to experimental tanks along with defluorinated brackish water (salinity of 10‰), with the temperature of the water being 24–30°C. The fish were acclimatized to laboratory conditions for two weeks before experiments. The fish used for study were healthy without any signs of illness, and the experiments were approved by the Committee on Animal Care and Use and the Committee on the Ethics of Animal Experiments of Ningbo University. Before acquisition of tissues and MO/MФ, we added 0.1% aqueous solution of tricaine (MS 222; Sigma-Aldrich, St. Louis, USA) to anesthetize the mudskippers. After the tissues were quickly excised, fish were euthanized by humane endpoints.

### Primary culture of mudskipper kidney-derived MO/MФ

Mudskipper kidney-derived MO/MФ were isolated as previously described [25]. In brief, kidney leukocyte-enriched fractions were obtained using Ficoll-Paque PREMIUM (GE healthcare, New Jersey, USA) density gradient centrifugation. Non-adherent cells were washed off, and the attached cells were incubated with a complete medium (RPMI 1640 medium containing 5% fetal calf serum, 5% mudskipper serum, 100 U/ml penicillin, and 100 μg/ml streptomycin) throughout the experiment after overnight incubation at 24°C. Following Giemsa staining, over 95% of the adherent cells were identified as MO/MФ, as confirmed by observing the morphological characteristics.

According to previously reported methods [26], mudskipper MO/MФ were maintained at 24°C in a 5% CO_2_ and 95% air incubator (21% O_2_, normoxia). Hypoxic cells were maintained at 24°C in a modular incubator chamber (Billups-Rothenberg, Del Mar, USA) flushed with a gas mixture containing 1% O_2_, 5% CO_2_, and 94% N_2_.

### Molecular cloning of BpHIF-1α cDNA

The cDNA sequence of BpHIF-1α gene was subsequently identified using transcriptome analysis. Total RNA was extracted from mudskippers using RNAiso reagent (TaKaRa, Dalian, China) following the manufacturer’s protocol. The first-strand of cDNA was synthesized with M-MLV reverse transcriptase (RNase H^−^; TaKaRa, Dalian, China) from a single cell. Sequence-specific primers were generated to amplify the BpHIF-1α cDNA (Table 1). The PCR amplification product was sequenced using an ABI 3730 automated sequencer (Invitrogen, Foster City, USA). Multiple sequence alignments were performed using ClustalW (http://clustalw.ddbj.nig.ac.jp/). The phylogenetic tree was generated by neighbor-joining analysis of HIF-1α proteins with bootstrapping of 1,000 replicates using MEGA version 5 [27]. The HIF-1α sequences used in this study are listed in Table 2.

**Table 1:** Oligonucleotide primers used in this study.

Primers	Nucleotide sequence (5′→3′)	Sequence information	Amplicon size (base pairs)
BpHIF-1α (+)	ATGGACGCTGGAGTCGT	PCR	2247
BpHIF-1α (-)	TCAGTTGACATGGTCCAGG	PCR	
BpHIF-1α-F	ATTTGCTAAGGGCCAGGTTT	RT-qPCR	151
BpHIF-1α-R	GCCACTCAGCACAAAGTTGA	RT-qPCR	
BpIL-1β-F	ACGAGTGGTGAATGTGGTCA	RT-qPCR	163
BpIL-1β-R	GAACTGAGGTTGTGCTGCAA	RT-qPCR	
BpIL-10-F	GTGGAGGGGTTCCCTCTAAG	RT-qPCR	179
BpIL-10-R	GTGCGGAGGTAAAAGCTCAG	RT-qPCR	
BpTGFβ-F	TCAAAGGACACTTGCACAGC	RT-qPCR	183
BpTGFβ-R	CAGGGCCAAGATCTGTGAAT	RT-qPCR	
BpTNFα-F	GGACAACAACGAGATCGTGA	RT-qPCR	155
BpTNFα-R	GTTCCACCGTGTGACTGATG	RT-qPCR	
*E*. *tarda* gyrB-F	TGGCGACACCGAGCAGA	RT-qPCR	207
*E*. *tarda* gyrB-R	ACAAACGCCTTAATCCCACC	RT-qPCR	
BpGlut1-F	CTTCGAGGTGCTCTGGGTAC	RT-qPCR	169
BpGlut1-R	GGCACAAAGGAAGCAAGACG	RT-qPCR	
BpVegfa-F	GCGCATATACCGAGGGAAGG	RT-qPCR	157
BpVegfa-R	CCACACAAGACGGGATGAAGA	RT-qPCR	
BpAldoc-F	GGAGAGACAACCACCCAAGG	RT-qPCR	165
BpAldoc-R	AGCGTATCTTGCCAGCACAT	RT-qPCR	
BpCSF1R-F	CACAGTGGAGGTGCAGAGAG	RT-qPCR	186
BpCSF1R-R	CCTCCAGCTCCGAACAAAGT	RT-qPCR	
BpiNOS-F	GCGAATGGGAGAGGGAGATG	RT-qPCR	160
BpiNOS-R	CTTCCAACTGCGGTCATTGC	RT-qPCR	
Bp18s rRNA-F	GGCCGTTCTTAGTTGGTGGA	RT-qPCR	112
Bp18s rRNA-R	CCCGGACATCTAAGGGCATC	RT-qPCR	

**Table 2:** HIF-1α sequences used for multiple sequence alignment and phylogenetic analyses.

Accession number	Species		Protein
	Latin name	English name	
KX278429	*Boleophthalmus pectinirostris*	mudskipper	HIF-1α
ABD32158	*Micropogonias undulatus*	Atlantic croaker	HIF-1α
XP_010732457	*Larimichthys crocea*	large yellow croaker	HIF-1α
XP_005477096	*Oreochromis mossambicus*	Mozambique tilapia	HIF-1α
NP_001296971	*Danio rerio*	zebrafish	HIF-1α
AFV39804	*Sparus aurata*	sea bream	HIF-1α
ACN10960	*Salmo salar*	Atlantic salmon	HIF-1α
ABV59209	*Cyprinus carpio*	common carp	HIF-1α
AAR95697	*Ctenopharyngodon idella*	grass carp	HIF-1α
ADF50043	*Megalobrama amblycephala*	Wuchang bream	HIF-1α
ADJ67806	*Hypophthalmichthys molitrix*	silver carp	HIF-1α
NP_001117760	*Oncorhynchus mykiss*	rainbow trout	HIF-1α
CAA64833	*Mus musculus*	mouse	HIF-1α
NP_001116596	*Sus scrofa*	pig	HIF-1α

### Preparation of antibody

Western blot for determining the expression of BpHIF-1α and Bpβ-actin, ELISA for measuring the expression of *B*. *pectinirostris* interleukin (IL)-10 and flow cytometry to determine the teleost macrophage marker (colony-stimulating factor 1 receptor, BpCSF1R) was performed. The products of the sequences mentioned in Table 3 were coupled to the carrier protein keyhole limpet hemocyanin to chemically synthesize for the preparation of the rabbit polyclonal antibody (GL Biochem, Shanghai, China).

**Table 3:** Amino acid sequence used for chemically synthesis.

Name of gene	Amino acid sequence
BpHIF-1α	^709^SSVLTLPQLTQYDCEVNAPLQGRQYLLQGEELLRALDHVN^748^
Bpβ-actin	^3^EEEIAALVVDNGSGMCKA^20^
BpIL-10	^29^CNNRCCRFVEGFPLRLRKLREDYSQIRD^56^
BpCSF1R	^700^PEIVENDYKNV^710^

### Bacterial infection and tissue sampling

To infect the fish specimens with *Edwardsiella tarda* (*E*. *tarda*), bacteria were cultured at 28°C in tryptose soya agar, and were harvested when they entered the logarithmic growth phase. Healthy mudskippers were divided into an infection group and a control group. Mudskippers were challenged *in vivo* by intraperitoneally injecting *E*. *tarda* (1 × 10^5^ colony-forming units (CFUs)/fish) in 100 μl phosphate-buffered saline (PBS) in the infected group, whereas only PBS was administered to the mudskippers in the control group. Liver, spleen, kidney, heart, gill, skin, intestine, and MO/MΦ samples were collected at 3, 6, 12, and 24 h post infection (hpi). Tissue and cell samples were collected from the two groups, immediately snap-frozen in liquid nitrogen, and preserved at −70°C until RNA extraction.

### Real-time quantitative PCR (RT-qPCR)

Total RNA was extracted from fish tissues and MO/MΦ were isolated using RNAiso reagents (TaKaRa, Dalian, China). Each RNA sample (5 μg) was incubated with 1 U DNase I (TaKaRa, Dalian, China) for 30 min at 37°C to remove residual genomic DNA. The first-strand of cDNA was synthesized using AMV reverse transcriptase (TaKaRa, Dalian, China). Gene-specific primers were designed based on cDNA fragments of BpHIF-1α, *B*. *pectinirostris* tumour necrosis factor α (BpTNFα), BpIL-1β, BpIL-10, *B*. *pectinirostris* transforming growth factor β (BpTGFβ), *B*. *pectinirostris* glucose transporter 1 (BpGlut1), *B*. *pectinirostris* vascular endothelial growth factor-a (BpVegf-a), *B*. *pectinirostris* aldolase C (BpAldoc), BpCSF1R, *B*. *pectinirostris* inducible nitric oxide synthase (BpiNOS), and Bp18S rRNA (Table 1). RT-qPCR was performed using SYBR premix Ex Taq II (Perfect Real Time; TaKaRa, Dalian, China). The master mixture was subjected to denaturation cycles for 300 s at 95°C, followed by 35 amplification cycles of 30 s at 95°C, 30 s at 60°C, and 30 s at 72°C in an ABI StepOne Real-Time PCR thermocycler (Applied Biosystems, Foster City, USA). The cycle threshold (Ct) values of BpHIF-1α and others for all samples were normalized to Bp18S rRNA expression using the 2^−ΔΔCt^ method. Tissue samples were collected from four fish for each group. MO/MФ samples were reproduced in four independent experiments.

### Anti-BpCSF1R IgG staining and flow cytometry

MO/MФ were washed in FACS buffer (PBS, 0.2% BSA, and 0.1% sodium azide) and were resuspended to a final concentration of 2 × 10^7^/ml. Cells (2 × 10^6^/ml) were incubated with 2 μl anti-BpCSF1R IgG or 2 μl isotype IgG for 30 min at 4°C. After washing, cells were incubated with the secondary antibody, donkey anti-mouse IgG-PerCP (Jackson ImmunoResearch Laboratories, Westgrove, USA) for 30 min at 4°C, washed, and analyzed by flow cytometry. Data were collected and analyzed on a Gallios Flow Cytometer (Beckman Coulter, Miami, USA).

### Western blot

BpHIF-1α protein expression was detected in the cell lysate of mudskipper MO/MФ after infection or hypoxia at 0, 3, 6, 12, and 24 h. Cells were washed twice in PBS and lysed in a buffer containing 20 mM HEPES, 1.5 mM MgCl_2_, 0.2 mM EDTA, 100 mM NaCl, 0.2 mM DTT, 0.5 mM sodium orthovanadate, 0.4 mM phenylmethylsulfonyl fluoride (pH 7.4), and Phosphatase Inhibitor Cocktail (Sigma Aldrich, St. Louis, USA). The protein concentration was measured in each soluble fraction using the Bradford method. For expression analysis, sodium dodecyl sulfate–polyacrylamide gel electrophoresis (SDS–PAGE), membrane transfer, antibody incubation, and electrochemiluminescence reaction were performed as previously described [28]. The optical density of the bands was quantified using the NIH ImageJ software.

### RNA interference

BpHIF-1α small interfering RNA (siRNA; 5′-CACCUGUGCCCUAUUUGGUGCUGAU-3′) and a scrambled siRNA sequence (5′-UUACGCUAUGAUAGCUGUCUCUGCU-3′) were designed and synthesized by Invitrogen. Transfection of cells with siRNA was performed using the Lipofectamine 2000 transfection reagent (Invitrogen, Carlsbad, USA) according to the manufacturer’s protocol. In brief, 5 μl of Lipofectamine 2000 in 250 μl of Opti-MEM (Invitrogen, Carlsbad, USA) was mixed with either 100 pmol BpHIF-1α siRNA or 100 pmol scrambled siRNA. The mixture was then incubated for 20 min at 25°C before adding it to MO/MФ with a final siRNA concentration of 40 nM. After a 5.5-h incubation period, the medium was replaced with the complete medium, and cells were cultured for another 48 h before collection. RT-qPCR was performed to confirm the mRNA levels after BpHIF-1α knockdown.

### Phagocytosis assay

Because the inhibitor topotecan is not highly HIF-specific [29], phagocytosis assays were performed with BpHIF-1α siRNA. As described previously [30], mudskipper kidney-derived MO/MФ were seeded in six-well plates (2 × 10^6^/ml), incubated with scrambled siRNA or BpHIF-1α siRNA, and were then subjected to hypoxia for another 3, 6, 12, and 24 h; normoxia-treated MO/MФ were used as the control. The cells were subsequently infected at a multiplicity of infection (MOI) of 20 with heat-killed *Escherichia coli* strain DH5α, which was labeled with fluorescein isothiocyanate (FITC; here after designated as FITC–DH5α) according to the manufacturer’s protocol. The cells were then washed three times with PBS on ice to remove extracellular bacteria. Trypan blue was used to quench the fluorescence outside of the cells. The fluorescence from intracellular bacteria in MO/MФ and other data were collected using a Gallios Flow Cytometer.

### Bactericidal activity assay

The bactericidal assay for mudskipper kidney-derived MO/MΦ was modified from a previously described method [31]. A standard curve was generated with the RT-qPCR results to assess the bacterial number. RNA obtained from *E*. *tarda* culture at a concentration of 10^7^ CFUs/ml was serially diluted 10-fold. Each RNA dilution was used to construct a standard curve. After pre-treatment with scrambled siRNA or BpHIF-1α siRNA, mudskipper MO/MФ (2 × 10^6^/ml) were infected with *E*. *tarda* at an MOI of 10 for 3, 6, 12, and 24 h post hypoxia or normoxia. Bacteria were incubated for 30 min at 24°C to enable bacterial attachment and internalization. Next, cells were treated with 100 μg/ml gentamicin (Invitrogen, Carlsbad, USA) to eradicate extracellular bacteria whilst maintaining the viability of the intracellular bacteria. One set of samples (uptake group) was washed extensively with PBS and lysed, and viable bacteria were subjected to RNA template preparation and RT-qPCR using primers *E*. *tarda* gyrB-F and gyrB-R (Table 1). To measure bactericidal activity, the remaining cells (kill group) were then washed and incubated with 12.5 μg/ml gentamicin for the indicated times. Cells were subjected to RNA template preparation and an RT-qPCR test using primers for the *E*. *tarda* gyrb gene. The Ct values obtained from RT-qPCR were used to calculate the total number of CFU/ml present in all the samples, based on the standard curve previously generated. Bacterial survival was determined by dividing the number of CFU in the kill group by those in the uptake group.

### Enzyme-linked immunosorbent assay (ELISA)

ELISA was performed for measuring BpIL-10 levels in the supernatant of MO/MФ as described previously [32]. Viable *E*. *tarda* cells were diluted in PBS at appropriate concentrations to infect mudskipper MO/MФ for different time periods at a MOI of 10 under hypoxic conditions, and the supernatant was collected for ELISA. Micro plates (Nunc ImmunoPlates, Rochester, USA) were coated using concentrated and quantified cell supernatants and incubated overnight. After the plates were precoated with poly-L-lysine (Sigma Aldrich, St. Louis, USA) to increase protein binding, blocking of unbound binding sites was performed using Tris-buffered saline with fish gelatin (Sigma Aldrich, St. Louis, USA). Anti-IL-10 antibody (IgG) was added to each well, incubated for 1 h, and washed three times with PBS. The secondary conjugated antibody was added to each well for 1 h and was washed three times. Finally, ELISA was performed using the alkaline phosphatase yellow liquid substrate system (Sigma Aldrich, St. Louis, USA) with the optical density set at 405 nm.

### Statistical analysis

All data are presented as mean ± standard error of the mean (SEM) [33]. The data were further analyzed by one-way ANOVA using SPSS version 13.0 (SPSS, Chicago, USA). In all cases, *P* < 0.05 was considered as statistically significant.

## Results

### Characterization of BpHIF-1α

**Fig 1:**
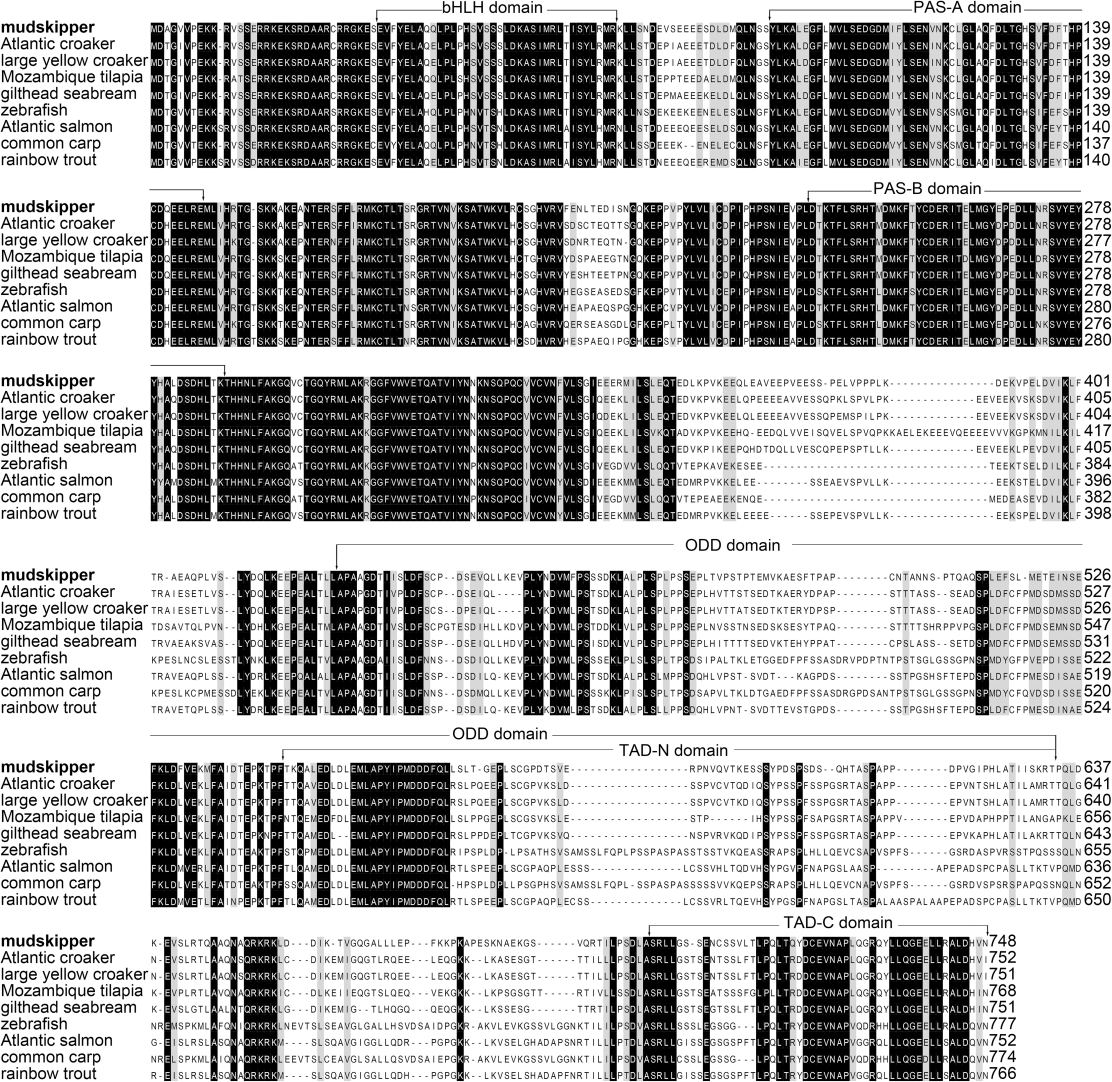
Alignment of the deduced amino acid sequence of mudskipper HIF-1α (Bold) with the same protein from other fishes. The alignment was generated using BioEdit version 7.2. Dashes indicate gaps introduced to facilitate alignment. The bHLH domain, PAS-A and PAS-B domains, ODD domain, and TAD-N and TAD-C domains are indicated.

BpHIF-1α sequence was deposited in the GenBank Data Library under accession number KX278429. The cDNA of BpHIF-1α possessed a large open reading frame of 2247 nucleotides that encoded a polypeptide of 748 amino acids. The molecular weight of the protein was 83.82 kDa and its putative isoelectric point was 4.83. BpHIF-1α and other teleost HIF-1α proteins showed relatively high homologies in the basic helix–loop–helix (bHLH, 85–98%), and the Per–ARNT–Sim A (PAS-A, 80–97%) and PAS-B (90–97%) domains. In contrast, two transactivation domains (TAD) and the oxygen-dependent degradation domain (ODD), particularly the N-terminal TAD (TAD-N) and ODD sequence, varied considerably among the different proteins, which were <40% identical in the TAD-N and ODD sequences (Fig 1).

**Fig 2:**
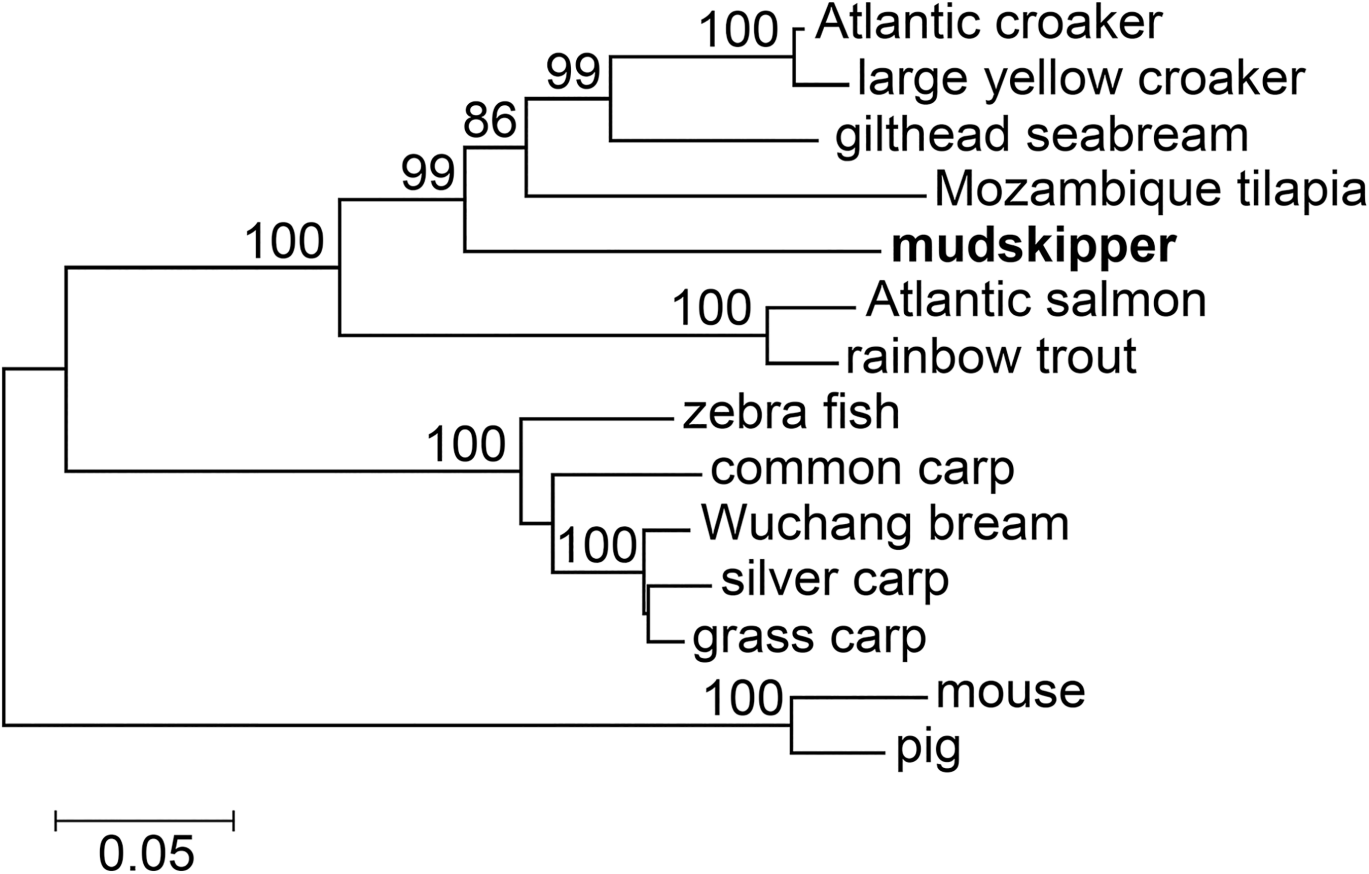
Phylogenetic tree comparing the sequence of mudskipper HIF-1α (Bold) with that of other vertebrate species. The scale bar refers to evolutionary distances in substitutions per site. The numbers at tree nodes refer to percentage bootstrap values after 1,000 replicates.

To analyze the evolutionary relationship of BpHIF-1α with respect to other publicly available related genes in other fish and mammalian species, a phylogenetic tree was constructed using the neighbor-joining method (Fig 2). Sequence comparisons for similarity showed that BpHIF-1α was highly similar to HIF-1α in fishes such as the Atlantic croaker (77.4%), followed by gilthead seabream (77%), and other fishes. BpHIF-1α belonged to the fish HIF-1α cluster with zebrafish HIF-1α, which is a genetic model fish species.

### Spatiotemporal expression of BpHIF-1α after challenging with infection

**Fig 3:**
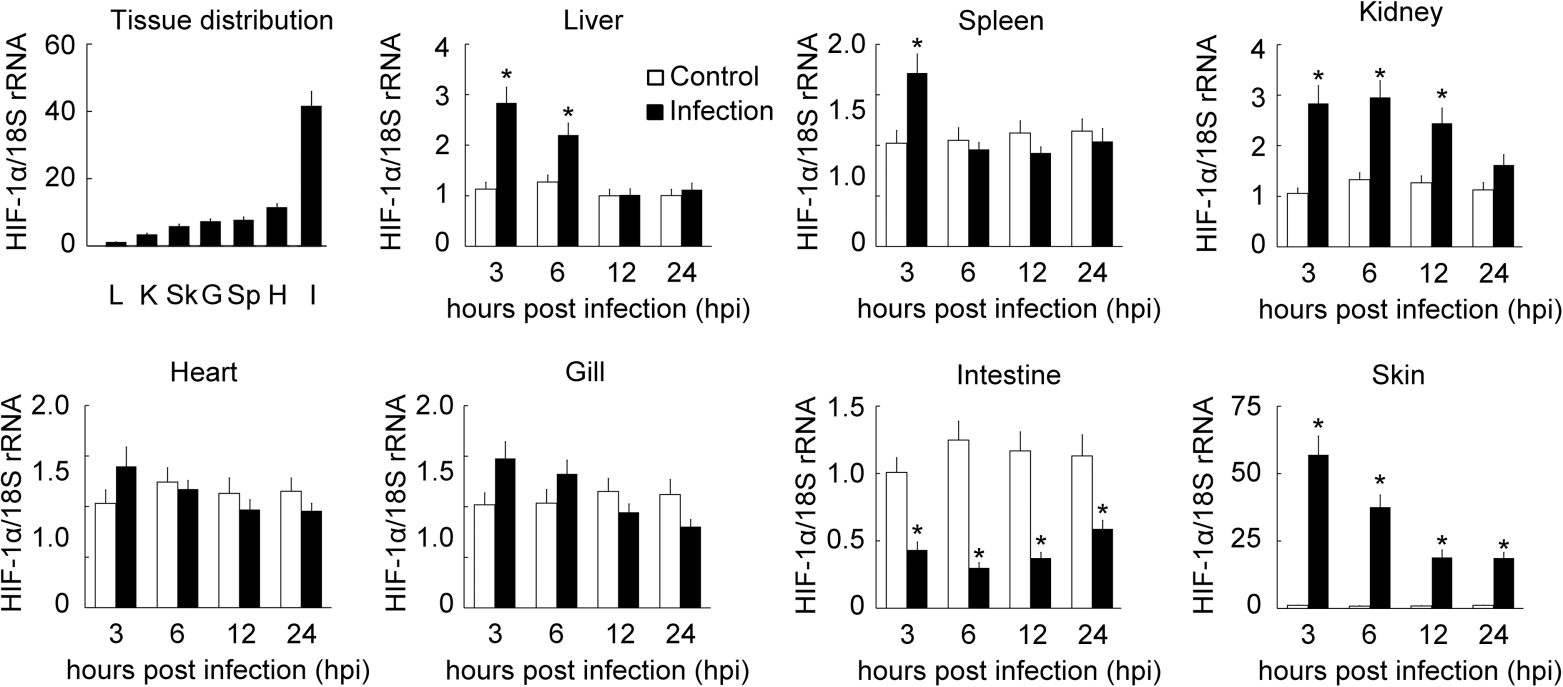
RT-qPCR analysis of HIF-1α mRNA expression in various mudskipper tissues. Fish were euthanized at 3, 6, 12, and 24 h after intraperitoneal injection of *E*. *tarda*, and PBS was used as the control. L, liver; Sp, spleen; K, kidney; H, heart; G, gill; I, intestine; Sk, skin. HIF-1α transcript levels were normalized to those of 18S rRNA. Data are expressed as mean ± SEM (n = 4 for tissues). *, *P*< 0.05.

To illustrate the relationship between infection and BpHIF-1α expression, RT-qPCR was performed to analyze the mRNA expression levels of BpHIF-1α in normal and infected tissues. As shown in Fig 3, the levels of constitutive BpHIF-1α transcript were measured in seven tissues from healthy mudskippers. BpHIF-1α transcripts were strongly expressed in the intestine, moderately expressed in the heart, spleen, kidney, skin, and gills, and weakly expressed in the liver. Following infection with *E*. *tarda*, BpHIF-1α transcripts were up-regulated in the skin at all tested time points, in the liver at 3 and 6 hpi, in the spleen at 3 hpi, and in the kidney at 3, 6, and 12 hpi. The up-regulation of BpHIF-1α mRNA was highest in the skin after being challenged with *E*. *tarda*, with the level being 57-fold higher than that in the control group at 3 hpi. BpHIF-1α mRNA expression was down-regulated in the intestine at all tested time points (Fig 3). Furthermore, BpHIF-1α transcript expression was not altered significantly in the heart and gill tissues following infection at any of the tested time points.

**Fig 4:**
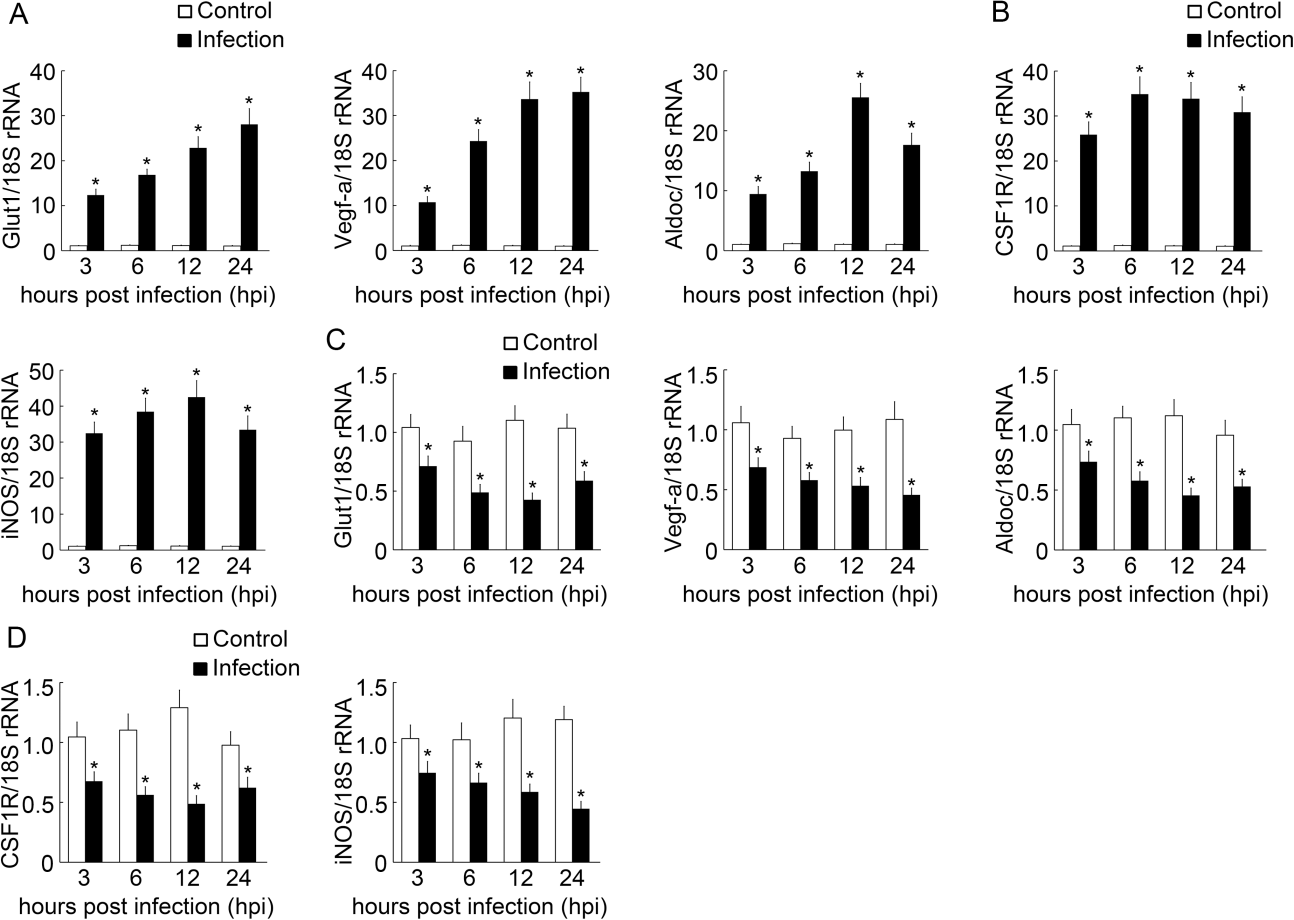
RT-qPCR analysis of mRNA expression in the skin and intestine after *E*. *tarda* infection. Fish were euthanized at 3, 6, 12, and 24 h after intraperitoneal injection of *E*. *tarda*, and PBS was used as the control. Then, the skin (A and B) and intestine (C and D) were collected. BpGlut1, BpVegf-a, BpAldoc, BpCSF1R, and BpiNOS transcript levels were normalized to those of 18S rRNA. Data are expressed as the mean ± SEM (n = 4 for tissues). *, *P*< 0.05.

To monitor BpHIF-1α activation and to explain spatiotemporal expression of BpHIF-1α mRNA in the skin and intestine after infection, we detected common HIF-1α target genes (BpGlut1, BpVegf-a, and BpAldoc), a marker of MO/MΦ in teleosts (BpCSF1R), and a macrophage inflammation marker (BpiNOS) mRNA expression in normal and infected tissues by RT-qPCR. Levels of BpGlut1, BpVegf-a, and BpAldoc transcripts were significantly up-regulated in the skin after infection at all tested time points (Fig 4A). BpCSF1R and BpiNOS mRNA expressions were up-regulated in the skin after infection (Fig 4B). On the contrary, mRNA levels of BpGlut1, BpVegf-a, BpAldoc, BpCSF1R, and BpiNOS were significantly down-regulated in the intestine after infection with *E*. *tarda* (Fig 4C and 4D).

**Fig 5:**
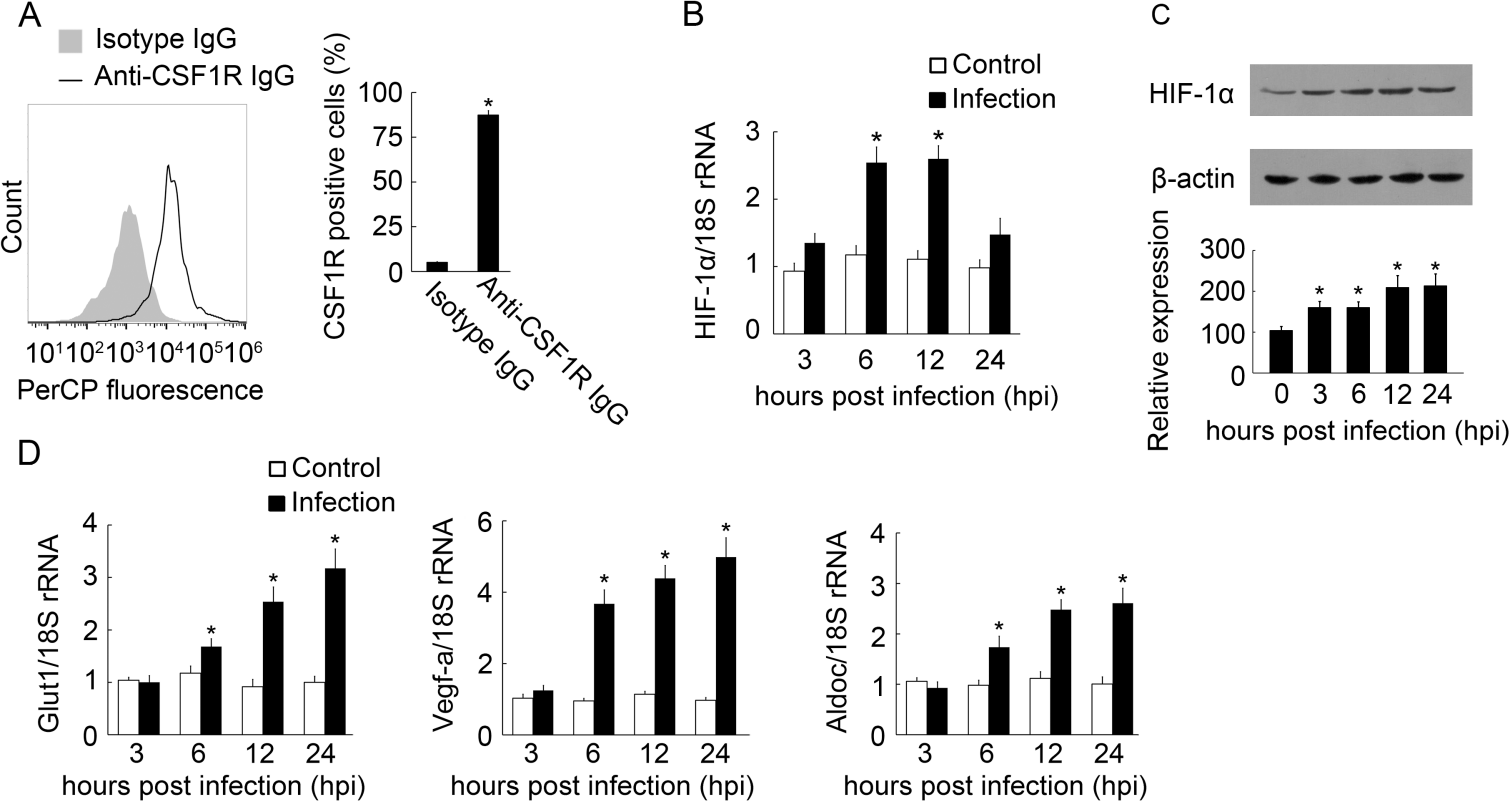
HIF-1α expression in MO/MΦ of mudskippers after infection with *E*. *tarda*. (A) Flow cytometry analysis of the percentage of CSF1R-positive cells in mudskipper kidney-derived MO/MΦ following incubation with anti-CSF1R IgG or isotype IgG. Histogram represents the percentage of CSF1R-positive cells in the anti-CSF1R IgG or isotype IgG-treated groups. (B and D) MO/ MΦ were collected at 3, 6, 12, and 24 hpi with *E*. *tarda*, and PBS was used as the control. The transcript levels of HIF-1α and HIF-1α target genes were normalized to those of 18S rRNA. Data are expressed as mean ± SEM for four independent experiments. *, *P* < 0.05. (C) Western blot analysis of mature BpHIF-1α protein in MO/MФ at 0, 3, 6, 12, and 24 hpi with *E*. *tarda*. Histogram displays the changes in relative band intensity of BpHIF-1α protein after infection. Quantification of BpHIF-1α protein expression is presented as fold change over β-actin, which was assigned a unit of 100. Data are representative of three independent experiments. *, *P* < 0.05.

To ascertain if BpHIF-1α expression was altered in MO/MФ after infection, RT-qPCR and western blot were performed to analyze the expression levels of BpHIF-1α in normal and infected MO/MФ. Firstly, flow cytometry was used to characterize kidney-derived MO/MФ, and 87.4% of the MO/MФ were found to be CSF1R-positive cells (Fig 5A). BpHIF-1α transcripts increased by 2.54- and 2.60- fold at 6 and 12 hpi, respectively (Fig 5B). Western blot analysis showed that BpHIF-1α protein in MO/MФ was up-regulated at 3, 6, 12, and 24 hpi (Fig 5C). All BpHIF-1α target genes (BpGlut1, BpVegf-a, and BpAldoc) were up-regulated at tested time points, except at 3 hpi (Fig 5D).

### BpHIF-1α expression in MO/MФ in response to hypoxia

**Fig 6:**
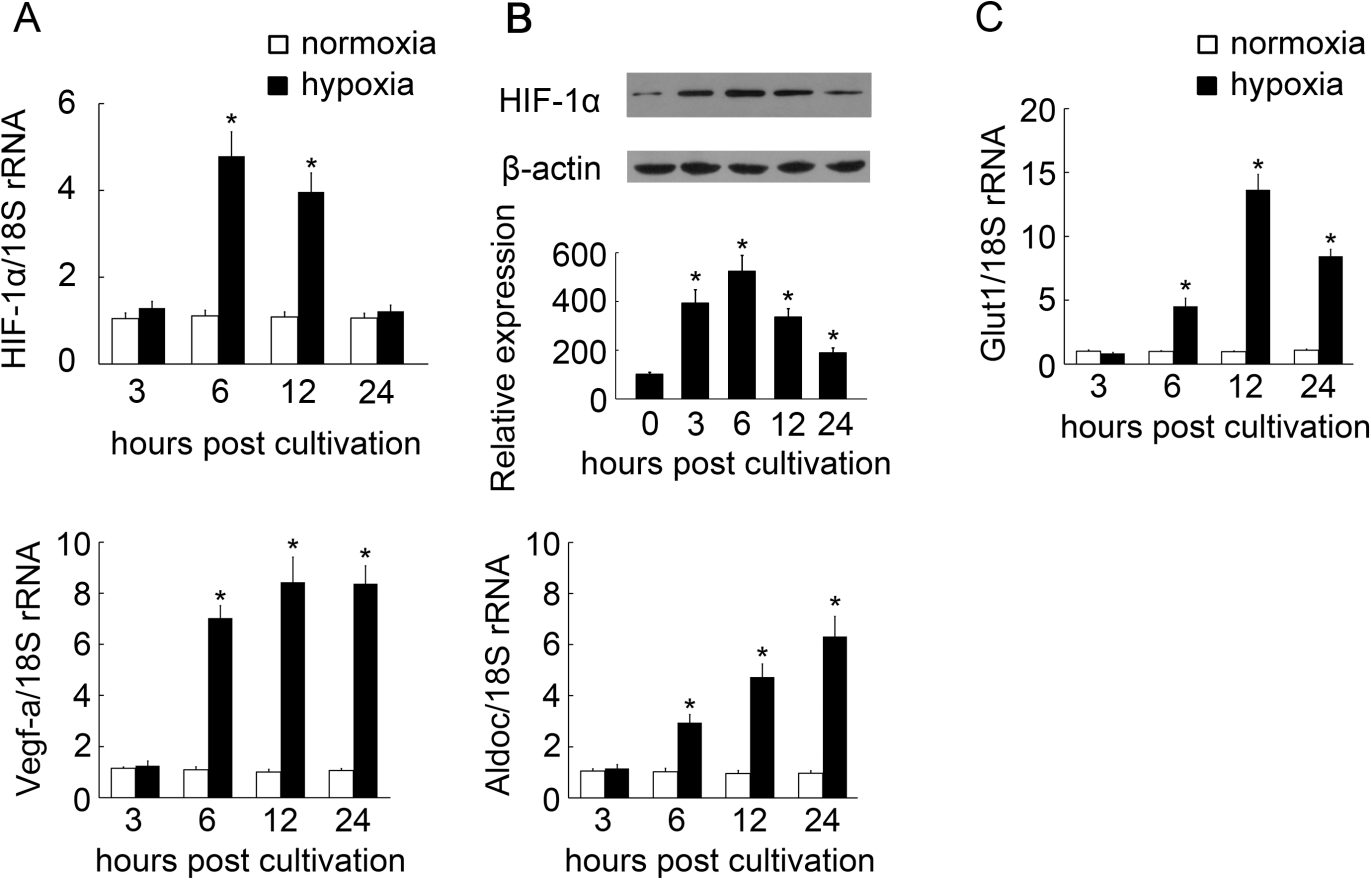
Alteration of HIF-1α expression in MO/MΦ of mudskippers cultured in parallel with either normoxia or hypoxia. (A and C) MO/ MΦ were collected at 3, 6, 12, and 24 h post normoxia or hypoxia, incubated in normoxia (21% O_2_) or in hypoxia (1% O_2_). The transcript levels of HIF-1α and HIF-1α target genes were normalized to those of 18S rRNA. Data are expressed as mean ± SEM for four independent experiments. *, *P* < 0.05. (B) Western blot analysis of mature BpHIF-1α protein in MO/MФ at 0, 3, 6, 12, and 24 h post hypoxia. Histogram displays the changes in relative band intensity of BpHIF-1α protein on hypoxia. Quantification of BpHIF-1α protein expression is presented as fold change over β-actin, which was assigned a unit of 100. Data are representative of three independent experiments. *, *P* < 0.05.

To ascertain whether BpHIF-1α expression was altered in MO/MФ under hypoxic conditions, we performed RT-qPCR and western blot. MO/MФ were cultured extensively and then exposed to ambient oxygen levels or hypoxia while maintaining identical oxygen concentration. We profiled global mRNA levels at 3, 6, 12, and 24 h post hypoxia. RT-qPCR confirmed that BpHIF-1α transcript levels were up-regulated 4- to 5-fold under hypoxia than under normoxia at 6 and 12 h (Fig 6A). Western blot analysis showed that BpHIF-1α protein in MO/MФ was up-regulated at 3, 6, 12, and 24 h post hypoxia (Fig 6B). BpHIF-1α target genes (BpGlut1, BpVegf-a, and BpAldoc) were induced at all tested time points, except at 3h after hypoxia (Fig 6C).

### Effect of hypoxia-induced BpHIF-1α activation on phagocytosis in MO/MФ

**Fig 7:**
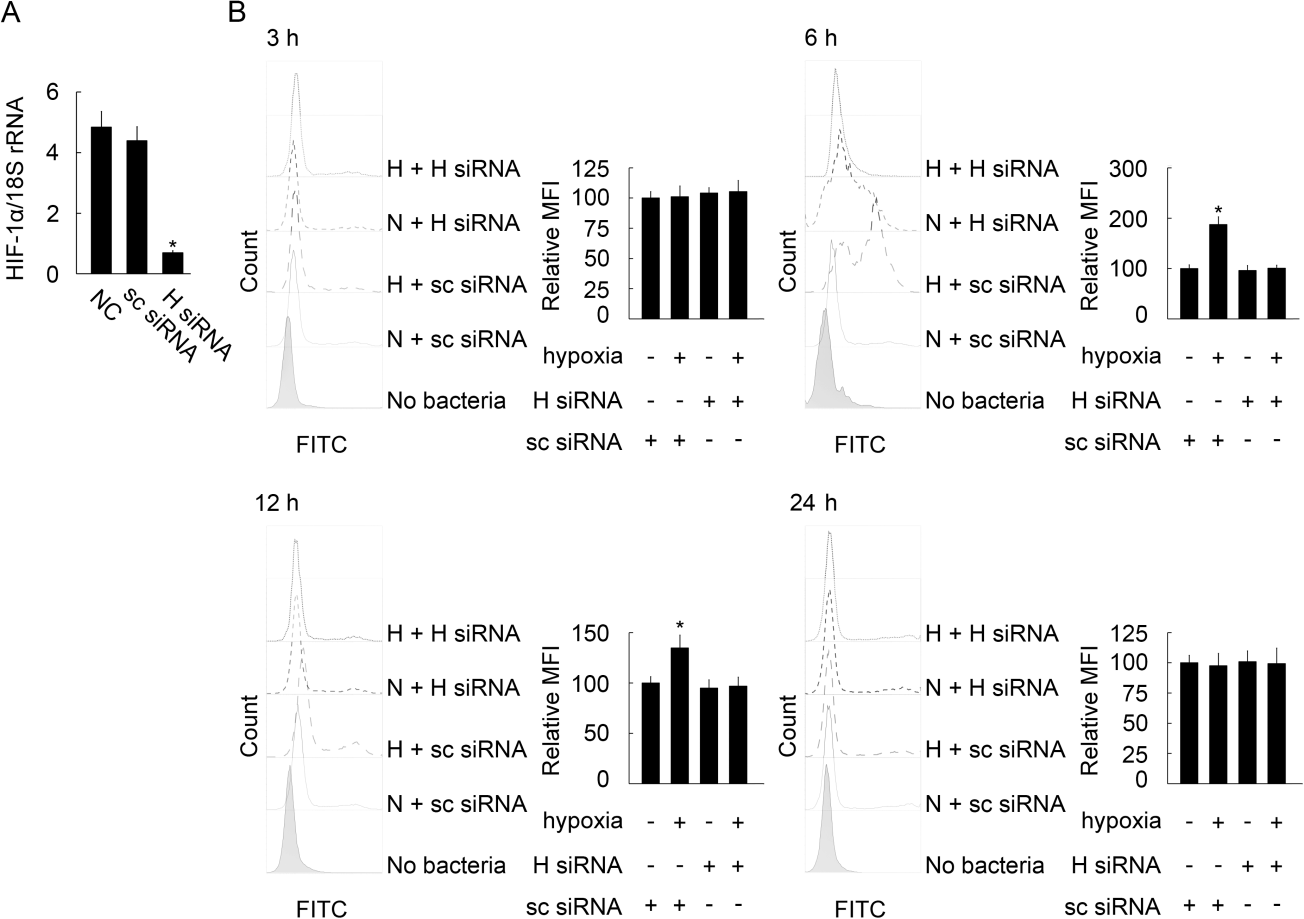
Hypoxia-influenced phagocytosis of mudskipper MO/MΦ. (A) Histogram displays the effect of BpHIF-1α siRNA transfection on MO/MΦ BpHIF-1α mRNA expression in a hypoxic environment, as detected by RT-qPCR. Transcript levels of BpHIF-1α were normalized to those of 18S rRNA. Data are expressed as mean ± SEM for four independent experiments. *, *P* < 0.05. (B) MO/MΦ were transfected with 100 pmol BpHIF-1α siRNA (H siRNA) or 100 pmol scrambled siRNA (sc siRNA) in 250 μl of Opti-MEM for the indicated length of time under normoxic (N, −) or hypoxic (H, +) conditions, respectively. Then, FITC–DH5α was added at an MOI of 20, followed by incubation for 1 h. The phagocytosed bacteria were investigated by flow cytometry assays. Quantification of phagocytosis was presented as fold change over the control, which was assigned a unit of 100. Data are expressed as mean ± SEM for three separate experiments with a total of 100,000 cells per experiment. *, *P*< 0.05.

Infectious diseases are often encountered in association with extracellular hypoxia [34]. We, therefore, investigated whether hypoxia induced an effect on the phagocytic activity of mudskipper MO/MФ. The following results were demonstrated: First, BpHIF-1α mRNA was dramatically down-regulated in BpHIF-1α-siRNA-treated MO/MФ after hypoxia (Fig 7A). Second, in scrambled siRNA-treated MO/MФ, hypoxia led to a significantly higher phagocytic activity of MO/MФ at 6 and 12 h post hypoxia compared with that post normoxia (Fig 7B). Thirdly, BpHIF-1α siRNA treatment led to lower phagocytic capacity of MO/MФ compared to treatment with scrambled siRNA at 6 and 12 h post hypoxia (Fig 7B).

### Effect of hypoxia-induced BpHIF-1α activation on bactericidal activity of MO/MФ

**Fig 8:**
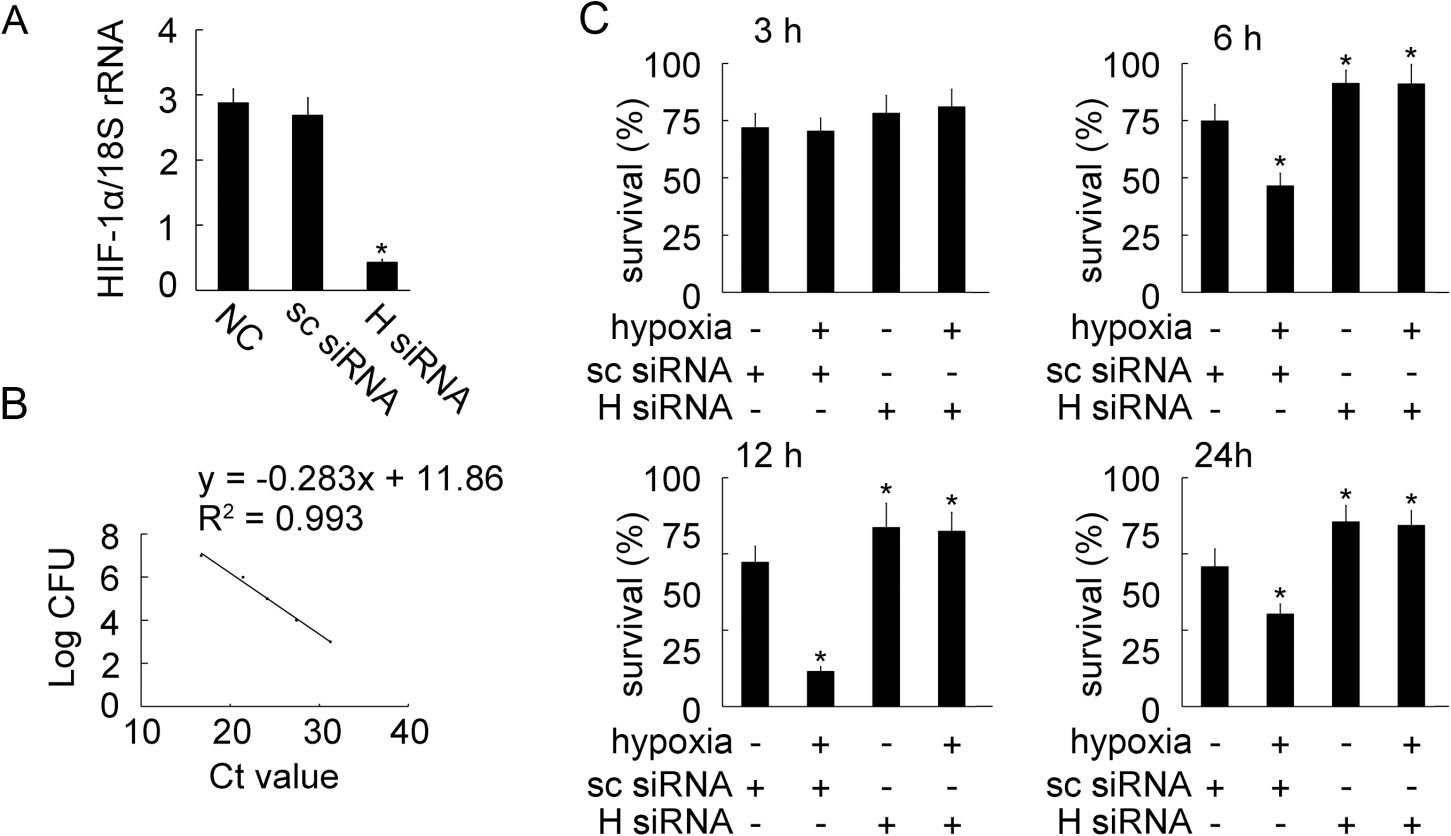
Effects of hypoxia on MO/MΦ bactericidal activity. Hypoxic MO/MΦ retained the ability to eradicate *E*. *tarda*. (A) Histogram displays the effect of BpHIF-1α siRNA transfection on MO/MΦ BpHIF-1α mRNA expression after infection, as detected by RT-qPCR. Transcript levels of BpHIF-1α were normalized to those of 18S rRNA. Data are expressed as mean ± SEM for four independent experiments. *, *P* < 0.05. (B) Standard curve was generated from 10-fold serial dilutions of *E*. *tarda*, as detected by RT-qPCR. (C) MO/MФ were transfected with scrambled siRNA or BpHIF-1α siRNA for the indicated length of time. *E*. *tarda* bacterial cells were added at an MOI of 10 and incubated for additional 30 min. Viable bacterial cells were enumerated by RT-qPCR, as described in Materials and Methods. Histograms represent the viability rate (%) of bacteria. Each bar represents mean ± SEM of the results from four independent experiments. *, *P* < 0.05.

We next determined whether BpHIF-1α mediates the effect of hypoxia on bactericidal activity. We performed RT-qPCR for BpHIF-1α siRNA, which showed a high knockdown efficiency (87.09%) in MO/MФ after infection (Fig 8A). To confirm the CFU of viable *E*. *tarda*, RT-qPCR was performed by diluting the purified total RNA from bacteria, and constructing a general standard curve (Fig 8B). After MO/MФ cultivation under hypoxic or normoxic conditions, the survival rate of the bacteria was determined by quantifying intracellular *E*. *tarda* CFU number in mudskipper MO/MФ. The hypoxic treatment improved the bactericidal capacity of MO/MФ at 6, 12, and 24 h post hypoxia. At these three time points post hypoxia, the bacterial survival rates were 42.78 ± 4.36%, 14.08 ± 1.57%, and 36.93 ± 3.24%, respectively (Fig 8C). This enhanced bactericidal activity was prevented by BpHIF-1α siRNA, and increased survival of bacteria was observed with BpHIF-1α siRNA compared to that with scrambled siRNA under hypoxic conditions (Fig 8C).

### Hypoxia-mediated effects on *E*. *tarda*-stimulated cytokine expression by MO/MФ via BpHIF-1α

**Fig 9:**
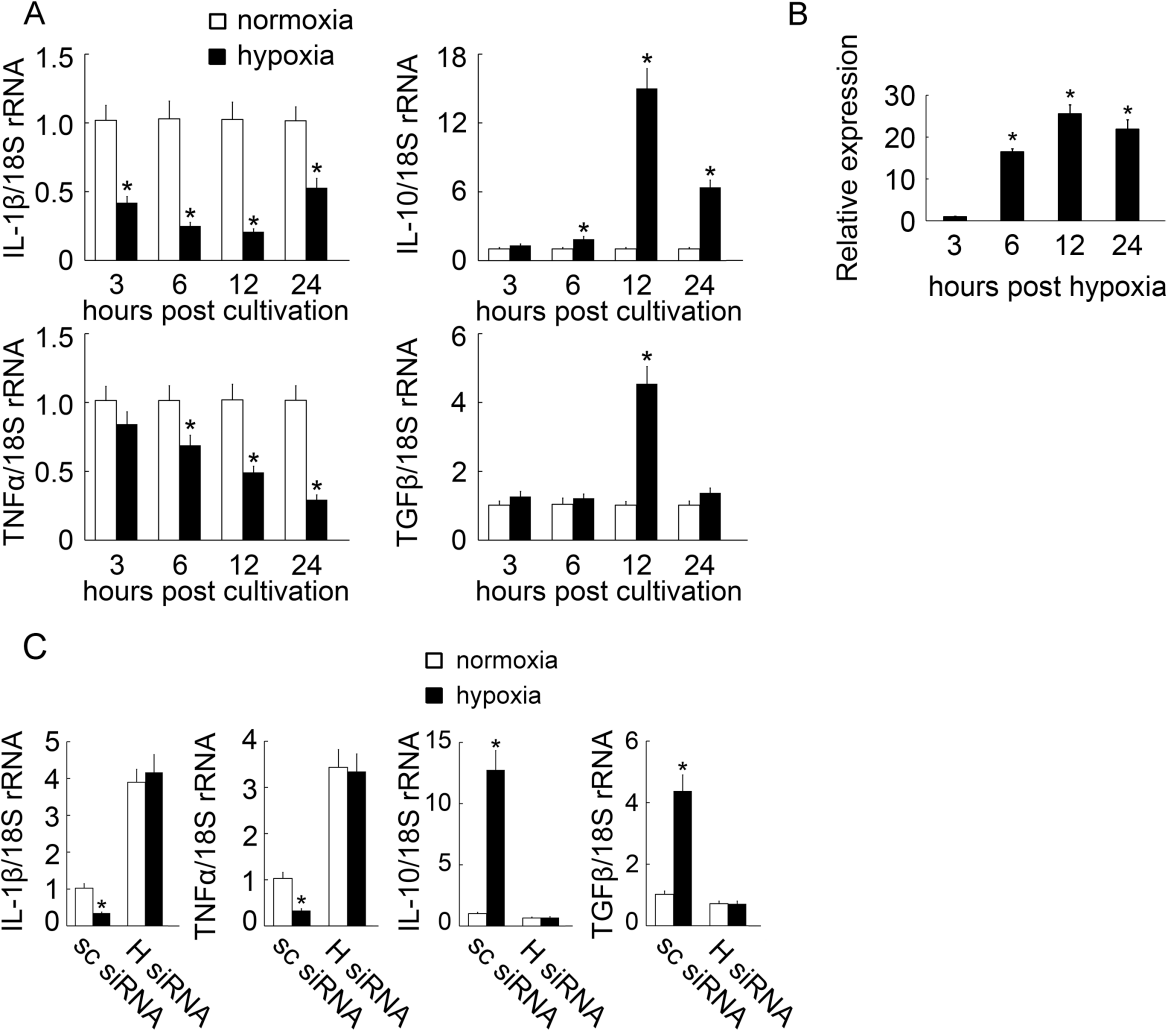
Effects of hypoxia on the expression of cytokines in mudskipper MO/MΦ challenged with viable *E*. *tarda* cells. MO/MΦ were incubated under normoxic or hypoxic conditions. *E*. *tarda* cells were added to the culture media at an MOI of 10. Normoxia-treated MO/MΦ were used as the control. (A) MO/MΦ were subjected to normoxia or hypoxia for 3, 6, 12, and 24 h. At each time point, BpIL-1β, BpTNFα, BpIL-10, or BpTGFβ transcript is presented as the fold change over the control, which was normalized to Bp18S rRNA using the 2^−ΔΔCt^ method. Data are expressed as mean ± SEM for four independent experiments. *, *P* < 0.05. (B) BpIL-10 protein expression was measured after *E*. *tarda* infection and hypoxia in MO/MΦ by ELISA. Quantification of BpIL-10 protein expression was presented as fold change over the value at 3 h, which was assigned a unit of 1. Data are expressed as mean ± SEM of the results from four independent experiments. *P < 0.05. (C) MO/MΦ were incubated with scrambled siRNA (sc siRNA) or BpHIF-1α siRNA (H siRNA) at 12 h post normoxia or hypoxia, respectively. Normoxia-treated MO/MΦ were used as the control. The cytokine transcript is presented as fold change over the control, which was normalized to Bp18S rRNA using the 2^−ΔΔCt^ method. Data are expressed as mean ± SEM for four independent experiments. *, *P* < 0.05.

To investigate whether hypoxia influences the expression of inflammatory cytokines BpIL-1β, BpTNFα, BpTGFβ, and BpIL-10, we determined the mRNA expression of these cytokines in *E*. *tarda*-infected MO/MФ after subjecting the cells to hypoxia or normoxia. After challenging with *E*. *tarda*, the hypoxia-treated group showed significantly down-regulated mRNA expression of BpIL-1β at all tested time points post hypoxia and down-regulated mRNA expression of BpTNFα at 6, 12, and 24 h post hypoxia (Fig 9A). BpIL-10 and BpTGFβ mRNA expression was significantly up-regulated at 6, 12, and 24 h post hypoxia, and at 12 h post hypoxia, respectively (Fig 9A). We further investigated BpIL-10 protein levels after bacterial challenge and hypoxic treatment in MO/MФ by ELISA. BpIL-10 protein levels in the supernatants of *E*. *tarda*-infected MO/MФ were up-regulated under hypoxic conditions at all tested time points (Fig 9B). Hypoxia down-regulated the mRNA expression of BpIL-1β and BpTNFα in scrambled siRNA-treated MO/MФ at 12 h post hypoxia compared to that post normoxia (Fig 9C). However, the mRNA levels of BpIL-10 and BpTGFβ in MO/MФ with scrambled siRNA were significantly up-regulated at 12 h post hypoxia compared to that post normoxia (Fig 9C). Compared to normoxia-treated BpHIF-1α siRNA, no change was observed in the expression levels of BpIL-1β, BpTNFα, BpIL-10, or BpTGFβ mRNA in BpHIF-1α siRNA-treated MO/MФ at 12 h post hypoxia (Fig 9C).

## Discussion

In the present study, BpHIF-1α was cloned from mudskipper, *B*. *pectinirostris*, MO/MФ cDNA. The BpHIF-1α protein consists of several functional s including bHLH domain, PAS domains, TAD domains, and an oxygen-dependendomaint degradation domain (ODD) domain. The bHLH domain mediates binding to the hypoxia-responsive DNA elements in the promoters or enhancers of HIF-1α target genes. The PAS domain mediates heterodimerization, i.e., binding to HIF-1β/Arnt. The ODD domain, with its two prolyl hydroxylation motifs (P561 in the mudskipper corresponds to P564 in humans, and P428 corresponds to P402), is crucial to hypoxic HIF-1α protein stabilization, which is the most important mechanism for HIF activation. In the TAD-C hydroxylation of asparagine 725 (N725), FIH regulates the transcriptional activity of TAD-C [10–12]. These three hydroxylation motifs are of utmost importance for the regulation of HIF activity in mammals and are highly conserved in fish as well. A phylogenetic tree analysis further confirmed BpHIF-1α as a member of the fish HIF-1α cluster.

HIF-1α is expressed in a ubiquitous pattern, although its expression levels vary highly among tissues in mammals [35]. In this study, BpHIF-1α transcripts were widely distributed in tissues and were particularly abundant in the intestine. A ubiquitous expression pattern of HIF-1α in tissues has been found in studies conducted on grass carp and Chinese suckers [36, 37]. Unlike BpHIF-1α, grass carp HIF-1α mRNA is highly expressed in the eyes and kidneys [37]. Chinese sucker HIF-1α transcripts are expressed primarily in the liver, ovary, and testis [36]. The distinctive tissue expression pattern of HIF-1α may reflect the adaptations of different fish species to the environment. Up-regulated expression of the HIF-1α transcript has been observed during bacterial infection [34]. HIF-1α transcripts expression in parenchymal and other non-immune cells do not respond to infection [38–41]. In our study, BpHIF-1α mRNA expression was up-regulated in the skin, liver, spleen, and kidney after challenging with *E*. *tarda*, whereas it was down-regulated in the intestine after infection. CSF1R mRNA and HIF-1α target gene expression were up-regulated in the skin and down-regulated in the intestine post infection. Thus, intraperitoneal infection may lead to evasion/emigration of MO/MФ from the intestine into the peritoneal cavity and may then enter into the systemic circulation and reach the central organs and into the skin. Our data suggested that the up-regulation or down-regulation of HIF-1α mRNA expression in tissues may be due to increased or decreased levels of MO/MФ in the tissues.

In mice, HIF-1α can activate the innate immune system under hypoxic conditions, particularly macrophages, promoting phagocytosis and stimulating the release of antimicrobial peptides and inflammatory cytokines [42]. Phagocytosis, the process by which microbial pathogens are engulfed, and bactericidal activity, the process by which infectious agents are eliminated by MO/MФ, represent the first line of defense against bacterial infection in the innate immune response of fish [43]. Our results showed that the exposure of mudskipper MO/MФ to hypoxia markedly increases phagocytosis and bactericidal activity, suggesting that hypoxia activates the MO/MФ function in teleosts. Moreover, it has been demonstrated that bactericidal activity of MФ can be inhibited by hypoxia [44]. However, we found that 1% oxygen enhanced the bactericidal activity of mudskipper MO/MФ, which is consistent with a previous report [45]. We also performed a bactericidal assay with 0.5% oxygen and found that it does not promote the bactericidal activity of MO/MФ. Thus, we speculated that 0.5% oxygen may reduce the production of reactive oxygen species (ROS), and 1% oxygen can elevate the bactericidal activity by ROS of mudskipper MO/MФ. Furthermore, we found that the enhancement of phagocytosis and bactericidal activity by hypoxia was prevented in MO/MФ treated with BpHIF-1α siRNA, suggesting that BpHIF-1α is the main mediator of the hypoxic effect for macrophage activation. Future investigations will focus on the mechanisms underlying HIF-1α participation in the activation of MO/MФ.

Mammalian macrophages synthesize the inflammatory cytokines IL-1, TNFα, IL-6, and IL-10 in response to persistent infections, which recruit immune cells to sustain chronic inflammation [46]. Expression of IL-1β mRNA in Nile Tilapia mononuclear leucocytes is down-regulated in response to hypoxia [47]. mRNA levels of IL-1β and TNF-α genes are down-regulated in rainbow trout after infection with *Aeromonas salmonicida* [48]. Bacterial or LPS challenges result in significantly increased transcripts levels of IL-10 in teleost head kidneys [49, 50]. In our study, IL-10 and TGFβ transcript expression levels was enhanced in *E*. *tarda*-infected mudskipper MO/MФ after subjecting to hypoxia compared to that after normoxia, suggesting that BpHIF-1α may contribute to the anti-inflammatory effects of MO/MФ. There is increasing evidence suggesting an anti-inflammatory role of HIF-1α in chronic diseases, but a multitude of studies on HIF-1α in mouse macrophages show that HIF-1α partially supports (by increasing glycolysis and thus, energy supply) pro-inflammatory activation and gene expression in murine macrophages [45, 51, 52]. Thus, HIF-1α activation and anti- and pro-inflammatory cytokines may be different in different animal species. In grass carp, rgcIL-10 and rgcTGFβ1 independently suppress LPS-stimulated pro-inflammatory gene expression in MO/MФ [53]. In this study, hypoxic BpHIF-1α down-regulated TNFα and IL-1β expression and up-regulated TGFβ and IL-10 expression in MO/MФ after bacterial challenge. Further studies are required to demonstrate whether interplay of pro-inflammatory and anti-inflammatory cytokines exists in hypoxia and inflammation induced in MO/MФ.

In summary, we identified a HIF-1α gene in the mudskipper. Upon *E*. *tarda* challenge, BpHIF-1α mRNA was significantly up-regulated in most tissues and MO/MФ. Moreover, *E*. *tarda* induced the expression of the BpHIF-1α protein in MO/MФ. Hypoxia-induced BpHIF-1α effect was enhanced in response to bacterial infection for regulating mudskipper MO/MФ function. Further studies are required to determine the detailed mechanisms underlying the role of HIF-1α in regulation of the immune response in fish.

## References

[1] Zhu CD, Wang ZH, Yan B. Strategies for hypoxia adaptation in fish species: a review. Journal of Comparative Physiology B. 2013;183(8): 1005–1013. 10.1007/s00360-013-0762-3 23660827

[2] Couturier CS, Stecyk JA, Rummer JL, Munday PL, Nilsson GE. Species-specific effects of near-future CO_2_ on the respiratory performance of two tropical prey fish and their predator. Comparative Biochemistry and Physiology Part A: Molecular & Integrative Physiology. 2013;166(3): 482–489. 10.1016/j.cbpa.2013.07.025 PMC3830952 23916817

[3] Domenici P, Herbert N, Lefrançois C, Steffensen JF, McKenzie D. The effect of hypoxia on fish swimming performance and behaviour Swimming physiology of fish. Springer; 2013 pp. 129–159.

[4] Wiegertjes GF, Wentzel AS, Spaink HP, Elks PM, Fink IR. Polarization of immune responses in fish: The ‘macrophages first’point of view. Molecular immunology. 2016;69: 146–156. doi: 10.1016/j.molimm.2015.09.026 26471699 10.1016/j.molimm.2015.09.026

[5] Ni M, Wen H, Li J, Chi M, Bu Y, Ren Y, et al The physiological performance and immune responses of juvenile Amur sturgeon (Acipenser schrenckii) to stocking density and hypoxia stress. Fish & shellfish immunology. 2014;36(2): 325–335. 24355406 10.1016/j.fsi.2013.12.002

[6] Kvamme BO, Gadan K, Finne-Fridell F, Niklasson L, Sundh H, Sundell K, et al Modulation of innate immune responses in Atlantic salmon by chronic hypoxia-induced stress. Fish & shellfish immunology. 2013;34(1): 55–65. 23085636 10.1016/j.fsi.2012.10.006

[7] Zhao Y, Jiang X, Kong X, Di G, Nie G, Li X. Effects of hypoxia on lysozyme activity and antioxidant defences in the kidney and spleen of *Carassius auratus*. Aquaculture Research. 2015.

[8] Li XF, Xu C, Tian HY, Jiang GZ, Zhang DD, Liu WB. Feeding rates affect stress and non-specific immune responses of juvenile blunt snout bream *Megalobrama amblycephala* subjected to hypoxia. Fish & shellfish immunology. 2016;49: 298–305. 26772476 10.1016/j.fsi.2016.01.004

[9] Semenza GL. Hypoxia-inducible factor 1: master regulator of O_2_ homeostasis. Current opinion in genetics & development. 1998;8(5): 588–594. 9794818 10.1016/s0959-437x(98)80016-6

[10] Epstein ACR, Gleadle JM, Mcneill LA, Hewitson KS, O'Rourke J, Mole DR, et al C. elegans EGL-9 and Mammalian Homologs Define a Family of Dioxygenases that Regulate HIF by Prolyl Hydroxylation. Cell. 2001;107(1): 43–54. 11595184 10.1016/s0092-8674(01)00507-4

[11] Jaakkola P, Mole DR, Tian YM, Wilson MI, Gielbert J, Gaskell SJ, et al Targeting of HIF-α to the von Hippel-Lindau ubiquitylation complex by O_2_-regulated prolyl hydroxylation. Science. 2001;292(5516): 468–472. doi: 10.1126/science.1059796 11292861 10.1126/science.1059796

[12] Masson N, Willam C, Maxwell PH, Pugh CW, Ratcliffe PJ. Independent function of two destruction domains in hypoxia-inducible factor-α chains activated by prolyl hydroxylation. Embo Journal. 2001;20(18): 5197–5206. doi: 10.1093/emboj/20.18.5197 11566883 10.1093/emboj/20.18.5197 PMC125617

[13] Iyer NV, Kotch LE, Agani F, Leung SW, Laughner E, Wenger RH, et al Cellular and developmental control of O_2_ homeostasis by hypoxia-inducible factor 1α. Genes & development. 1998;12(2): 149–162. 9436976 10.1101/gad.12.2.149 PMC316445

[14] Frede S, Stockmann C, Freitag P, Fandrey J. Bacterial lipopolysaccharide induces HIF-1 activation in human monocytes via p44/42 MAPK and NF-κB. Biochemical Journal. 2006;396(3): 517–527. doi: 10.1042/BJ20051839 16533170 10.1042/BJ20051839 PMC1482811

[15] Terova G, Rimoldi S, Corà S, Bernardini G, Gornati R, Saroglia M. Acute and chronic hypoxia affects HIF-1α mRNA levels in sea bass (Dicentrarchus labrax). Aquaculture. 2008;279(1): 150–159.

[16] Rahman MS, Thomas P. Molecular cloning, characterization and expression of two hypoxia-inducible factor alpha subunits, HIF-1α and HIF-2α, in a hypoxia-tolerant marine teleost, Atlantic croaker (*Micropogonias undulatus*). Gene. 2007;396(2): 273–282. doi: 10.1016/j.gene.2007.03.009 17467194 10.1016/j.gene.2007.03.009

[17] Sollid J, Rissanen E, Tranberg HK, Thorstensen T, Vuori KA, Nikinmaa M, et al HIF-1α and iNOS levels in crucian carp gills during hypoxia-induced transformation. Journal of Comparative Physiology B. 2006;176(4): 359–369. 10.1007/s00360-005-0059-2 16362306

[18] Fujisaka S, Usui I, Ikutani M, Aminuddin A, Takikawa A, Tsuneyama K, et al Adipose tissue hypoxia induces inflammatory M1 polarity of macrophages in an HIF-1α-dependent and HIF-1α-independent manner in obese mice. Diabetologia. 2013;56(6): 1403–1412. doi: 10.1007/s00125-013-2885-1 23494472 10.1007/s00125-013-2885-1

[19] Hagberg H, Gilland E, Bona E, Hanson LA, Hahinzoric M, Blennow M, et al Enhanced expression of interleukin (IL)-1 and IL-6 messenger RNA and bioactive protein after hypoxia-ischemia in neonatal rats. Pediatric Research. 1996;40(4): 603–609. doi: 10.1203/00006450-199610000-00015 8888290 10.1203/00006450-199610000-00015

[20] Anand RJ, Gribar SC, Li J, Kohler JW, Branca MF, Dubowski T, et al Hypoxia causes an increase in phagocytosis by macrophages in a HIF-1α-dependent manner. Journal of leukocyte biology. 2007;82(5): 1257–1265. doi: 10.1189/jlb.0307195 17675562 10.1189/jlb.0307195

[21] Wiese M, Gerlach RG, Popp I, Matuszak J, Mahapatro M, Castiglione K, et al Hypoxia-mediated impairment of the mitochondrial respiratory chain inhibits the bactericidal activity of macrophages. Infection and immunity. 2012;80(4): 1455–1466. doi: 10.1128/IAI.05972-11 22252868 10.1128/IAI.05972-11 PMC3318416

[22] Peyssonnaux C, Cejudo-Martin P, Doedens A, Zinkernagel AS, Johnson RS, Nizet V. Cutting edge: essential role of hypoxia inducible factor-1α in development of lipopolysaccharide-induced sepsis. The Journal of Immunology. 2007;178(12): 7516–7519. 17548584 10.4049/jimmunol.178.12.7516

[23] Walmsley SR, Chilvers ER, Whyte MK. Hypoxia, hypoxia inducible factor and myeloid cell function. Arthritis Research and Therapy. 2009;11: 219–225. doi: 10.1186/ar2632 19435530 10.1186/ar2632 PMC2688173

[24] You X, Bian C, Zan Q, Xu X, Liu X, Chen J, et al Mudskipper genomes provide insights into the terrestrial adaptation of amphibious fishes. Nature communications. 2014;5: 5594 doi: 10.1038/ncomms6594 25463417 10.1038/ncomms6594 PMC4268706

[25] Zhang R, Chen J, Li C, Lu X, Shi Y. Prokaryotic expression, purification, and refolding of leukocyte cell-derived chemotaxin 2 and its effect on gene expression of head kidney-derived macrophages of a teleost fish, ayu (Plecoglossus altivelis). Fish & shellfish immunology. 2011;31(6): 911–918. 21871568 10.1016/j.fsi.2011.08.008

[26] Mi Z, Rapisarda A, Taylor L, Brooks A, Creighton-Gutteridge M, Melillo G, et al Synergystic induction of HIF-1α transcriptional activity by hypoxia and lipopolysaccharide in macrophages. Cell Cycle. 2008;7(2): 232–241. doi: 10.4161/cc.7.2.5193 18212534 10.4161/cc.7.2.5193

[27] Tamura K, Peterson D, Peterson N, Stecher G, Nei M, Kumar S. MEGA5: molecular evolutionary genetics analysis using maximum likelihood, evolutionary distance, and maximum parsimony methods. Molecular biology and evolution. 2011;28(10): 2731–2739. doi: 10.1093/molbev/msr121 21546353 10.1093/molbev/msr121 PMC3203626

[28] Lu XJ, Chen X, Weng J, Zhang H, Pak D, Luo J, et al Hippocampal spine-associated Rap-specific GTPase-activating protein induces enhancement of learning and memory in postnatally hypoxia-exposed mice. Neuroscience. 2009;162(2): 404–414. doi: 10.1016/j.neuroscience.2009.05.011 19442707 10.1016/j.neuroscience.2009.05.011 PMC3243647

[29] Tsang PS, Cheuk AT, Chen QR, Song YK, Badgett TC, Wei JS, et al Synthetic Lethal Screen Identifies NF-κB as a Target for Combination Therapy with Topotecan for patients with Neuroblastoma. BMC Cancer. 2012;12(1): 101. 22436457 10.1186/1471-2407-12-101 PMC3364855

[30] Chen Q, Lu XJ, Li MY, Chen J. Molecular cloning, pathologically-correlated expression and functional characterization of the colonystimulating factor 1 receptor (CSF-1R) gene from a teleost, Plecoglossus altivelis. Zoological Research. 2016;37(2): 96–102. doi: 10.13918/j.issn.2095-8137.2016.2.96 27029867 10.13918/j.issn.2095-8137.2016.2.96 PMC4876830

[31] Yang GJ, Lu XJ, Chen Q, Chen J. Molecular characterization and functional analysis of a novel C-type lectin receptor-like gene from a teleost fish, Plecoglossus altivelis. Fish & shellfish immunology. 2015;44(2): 603–610. 25842180 10.1016/j.fsi.2015.03.037

[32] Tingbø MG, Pedersen ME, Grøndahl F, Kolset SO, Veiseth-Kent E, Enersen G, et al Type of carbohydrate in feed affects the expression of small leucine-rich proteoglycans (SLRPs), glycosaminoglycans (GAGs) and interleukins in skeletal muscle of Atlantic cod (Gadus morhua L.). Fish & shellfish immunology. 2012;33(3): 582–589. 22789715 10.1016/j.fsi.2012.06.025

[33] Lu XJ, Chen Q, Rong YJ, Yang GJ, Li CH, Xu NY, et al LECT2 drives haematopoietic stem cell expansion and mobilization via regulating the macrophages and osteolineage cells. Nature Communications. 2016;7: 12719 doi: 10.1038/ncomms12719 27596364 10.1038/ncomms12719 PMC5025878

[34] Zinkernagel AS, Johnson RS, Nizet V. Hypoxia inducible factor (HIF) function in innate immunity and infection. Journal of molecular medicine. 2007;85(12): 1339–1346. doi: 10.1007/s00109-007-0282-2 18030436 10.1007/s00109-007-0282-2

[35] Talks KL, Turley H, Gatter KC, Maxwell PH, Pugh CW, Ratcliffe PJ, et al The expression and distribution of the hypoxia-inducible factors HIF-1α and HIF-2α in normal human tissues, cancers, and tumor-associated macrophages. The American journal of pathology. 2000;157(2): 411–421. 10934146 10.1016/s0002-9440(10)64554-3 PMC1850121

[36] Chen N, Chen LP, Zhang J, Chen C, Wei XL, Gul Y, et al Molecular characterization and expression analysis of three hypoxia-inducible factor alpha subunits, HIF-1α/2α/3α of the hypoxia-sensitive freshwater species, Chinese sucker. Gene. 2012;498(1): 81–90. doi: 10.1016/j.gene.2011.12.058 22342256 10.1016/j.gene.2011.12.058

[37] Law SH, Wu RS, Ng PK, Richard M, Kong RY. Cloning and expression analysis of two distinct HIF-alpha isoforms-gcHIF-1alpha and gcHIF-4alpha-from the hypoxia-tolerant grass carp, *Ctenopharyngodon idellus*. BMC molecular biology. 2006;7(1): 1. 16623959 10.1186/1471-2199-7-15 PMC1473195

[38] Legendre C, Reen FJ, Mooij MJ, Mcglacken GP, Adams C, O'Gara F. Pseudomonas aeruginosa Alkyl quinolones repress hypoxia-inducible factor 1 (HIF-1) signaling through HIF-1α degradation. Infection & Immunity. 2012;80(11): 3985–3992. 22949552 10.1128/IAI.00554-12 PMC3486049

[39] Sharma M, Machuy N, Böhme L, Karunakaran K, Mäurer AP, Meyer TF, et al HIF-1α is involved in mediating apoptosis resistance to Chlamydia trachomatis-infected cells. Cellular Microbiology. 2011;13(10): 1573–1585. doi: 10.1111/j.1462-5822.2011.01642.x 21824245 10.1111/j.1462-5822.2011.01642.x

[40] Wiley M, Sweeney KR, Chan DA, Brown KM, Mcmurtrey C, Howard EW, et al Toxoplasma gondii activates hypoxia-inducible factor (HIF) by stabilizing the HIF-1alpha subunit via type I activin-like receptor kinase receptor signaling. Journal of Biological Chemistry. 2010;285(35): 26852–26860. doi: 10.1074/jbc.M110.147041 20581113 10.1074/jbc.M110.147041 PMC2930684

[41] Ng KT, Li JP, Ng KM, Tipoe GL, Leung WK, Fung ML. Expression of hypoxia-inducible factor-1alpha in human periodontal tissue. J Periodontol. 2011;82(1): 136–141. doi: 10.1902/jop.2010.100100 21043802 10.1902/jop.2010.100100

[42] Nizet V, Johnson RS. Interdependence of hypoxic and innate immune responses. Nature Reviews Immunology. 2009;9(9): 609–617. doi: 10.1038/nri2607 19704417 10.1038/nri2607 PMC4343208

[43] Ellis A. Immunity to bacteria in fish. Fish & shellfish immunology. 1999;9(4): 291–308.

[44] Wiese M, Gerlach RG, Popp I, Matuszak J, Mahapatro M, Castiglione K, et al Hypoxia-Mediated Impairment of the Mitochondrial Respiratory Chain Inhibits the Bactericidal Activity of Macrophages. 2012;80(4): 1455–1466. doi: 10.1128/IAI.05972-11 22252868 10.1128/IAI.05972-11 PMC3318416

[45] Peyssonnaux C, Datta V, Cramer T, Doedens A, Theodorakis EA, Gallo RL, et al HIF-1α expression regulates the bactericidal capacity of phagocytes. Journal of Clinical Investigation. 2005;115(7): 1806–1815. doi: 10.1172/JCI23865 16007254 10.1172/JCI23865 PMC1159132

[46] Wynn TA, Chawla A, Pollard JW. Macrophage biology in development, homeostasis and disease. Nature. 2013;496(7446): 445–455. doi: 10.1038/nature12034 23619691 10.1038/nature12034 PMC3725458

[47] Choi K, Lehmann D, Harms C, Law J. Acute hypoxia-reperfusion triggers immunocompromise in Nile tilapia. Journal of aquatic animal health. 2007;19(2): 128–140. doi: 10.1577/H06-010.1 18201054 10.1577/H06-010.1

[48] Mulder I, Wadsworth S, Secombes C. Cytokine expression in the intestine of rainbow trout (*Oncorhynchus mykiss*) during infection with *Aeromonas salmonicida*. Fish & shellfish immunology. 2007;23(4): 747–759. 17434320 10.1016/j.fsi.2007.02.002

[49] Chen J, Chen Q, Lu XJ, Li CH. LECT2 improves the outcomes in ayu with *Vibrio anguillarum* infection via monocytes/macrophages. Fish & shellfish immunology. 2014;41(2): 586–592. 25462453 10.1016/j.fsi.2014.10.012

[50] Seppola M, Larsen AN, Steiro K, Robertsen B, Jensen I. Characterisation and expression analysis of the interleukin genes, IL-1β, IL-8 and IL-10, in Atlantic cod (*Gadus morhua* L.). Molecular immunology. 2008;45(4): 887–897. doi: 10.1016/j.molimm.2007.08.003 17875325 10.1016/j.molimm.2007.08.003

[51] Cramer T, Yamanishi Y, Clausen BE, Förster I, Pawlinski R, Mackman N, et al HIF-1α Is Essential for Myeloid Cell-Mediated Inflammation. Cell. 2003;112(5): 645–657. 12628185 10.1016/s0092-8674(03)00154-5 PMC4480774

[52] Fang HY, Hughes R, Murdoch C, Coffelt S, Biswas SK, Harris AL, et al Hypoxia inducible factors 1 and 2 are important transcriptional effectors in primary macrophages experiencing hypoxia. Blood. 2009;114(114): 844–859. 19454749 10.1182/blood-2008-12-195941 PMC2882173

[53] Wei H, Yin L, Feng S, Wang X, Yang K, Zhang A, et al Dual-parallel inhibition of IL-10 and TGF-β1 controls LPS-induced inflammatory response via NF-κB signaling in grass carp monocytes/macrophages. Fish & shellfish immunology. 2015;44(2): 445–452. 25804490 10.1016/j.fsi.2015.03.023

